# Endothelial nitric oxide synthase gene polymorphisms and cardiovascular damage in hypertensive subjects: an Italian case-control study

**DOI:** 10.1186/1742-4933-5-4

**Published:** 2008-05-29

**Authors:** Daniela Colomba, Giovanni Duro, Salvatore Corrao, Christiano Argano, Tiziana Di Chiara, Domenico Nuzzo, Federica Pizzo, Gaspare Parrinello, Rosario Scaglione, Giuseppe Licata

**Affiliations:** 1 Department of Internal Medicine, University of Palermo, Italy; 2 Institute of Biomedicine and Molecular Immunology CNR, Palermo, Italy

## Abstract

**Background:**

Nitric oxide (NO) synthesized by endothelial nitric oxide synthase (eNOS) plays an important role in regulation of endothelial function and in the control of blood pressure. However, the results from some studies on the association between three clinically relevant eNOS gene polymorphisms (G894T, T786C and intron 4b/a) and essential hypertension are unclear. We designed a case-control study to evaluate the influence of eNOS polymorphisms on target organ damage in 127 hypertensives and 67 normotensives. Clinical evaluation, biochemical parameters, Urinary Albumin Excretion (UAE) and echocardiogram were performed to characterize target organ damage. eNOS polymorphism were recognized by PCR method.

**Results:**

The distribution of eNOS genotypes was similar in hypertensives and normotensives but 4aa was present in the 2.5% of hypertensives and completely absent in normotensives. Subjects with 4bb, G894T, and T786C genotypes showed an increased prevalence of target organ damage. Moreover prevalence of G894T and introne 4 variants was significantly higher in hypertensives than in normotensives both with cardiovascular damage. Logistic regression analysis didn't show any association between eNOS polymorphisms, Body Mass Index (BMI), hypertension, gender and cardiovascular damage. Only the age (OR 1.11; IC 95% 1.06–1.18) was predictive of cardiovascular damage in our population.

**Conclusion:**

Our results seem to indicate a lack of association with eNOS variants and cardiovascular damage onset.

## Background

Hypertension is a multifactorial disease involving both environmental and genetic components. Clinical and experimental studies suggest that an alteration in nitric oxide (NO) metabolism may be a contributing factor in the pathogenesis of hypertension. Thus, abnormalities in the activity of the enzyme endothelial NO synthase (eNOS) that synthesizes NO in endothelial cells may lead to NO deficiency with severe consequences. In fact, NO plays a pivotal role in the preservation of the endothelium homeostasis, regulation of vasomotor tone and control of blood pressure [1]

The gene encoding eNOS is located on chromosome 7 (7q35-q36) and contains 26 exons with an entire length of 21 kb. Disruption of eNOS gene leads to hypertension in mice [2] and inhibition of eNOS elevates blood pressure in healthy humans [3]. Furthermore, NO production is diminished in patients with essential hypertension (EH), under basal conditions [4]. On this important evidence the eNOS gene has been investigated as a putative candidate gene for EH. G894T, T786C and intron 4 (4b/a) are the most clinically relevant polymorphisms in the eNOS gene that have been described. However, the results of most association studies were inconsistent among different ethnicities and even among different populations from the same ethnicity [5-7].

The single nucleotide polymorphism (SNP), G894T within exon 7, resulting in a replacement of glutamic acid by aspartic acid at codon 298 (Glu298Asp), is the most highlighted variant whose association with hypertension is still controversial. In particular, the G894T was found to be associated with increased risk of hypertension in Caucasian [8] and Japanese [9], but in another two studies it was not (neither in Caucasian nor in Japanese) [10,11]. Recently, a functional 27-bp variable number of tandem repeats (VNTR) in intron 4 (intron4b/a) of eNOS gene has been reported to coordinate with another SNP at the promoter region (T786C). It regulates transcription efficiency in a haplotype-specific way which is modifiable by cigarette smoking [12]. Although no association has been reported between intron4b/a, T-786C and hypertension, most association studies did not examine the combining effect of these two polymorphisms on the development of hypertension [9,13,14]. Nonetheless, it is generally accepted that these variants can modify the NO production and may predispose non only to hypertension [5] but also to ischemic heart disease [15] or renal damage [16].

The aim of the present study was to investigate the relationship between eNOS polymorphisms, G894T, T786C and intron4b/a and cardiovascular damage in hypertensive patients. The main goal of the study was to evaluate if eNOS polymorphisms might be associated to cardiovascular damage in these subjects.

## Methods

We enrolled 127 subjects aged from 40 to 55 years, screened at the Department of Internal Medicine, University of Palermo (Italy). Subjects were considered to be hypertensive if their systolic/diastolic blood pressure was ≥ 140/90 mm Hg, confirmed in three following measurements; or ≥ 130/85 mm Hg if diabetic; or if the subjects were on hypertensive therapy. A mercury sphygmomanometer was used with cuff of suitable dimensions in all the obese subjects. The degree of obesity was assessed through the Body Mass Index (BMI) and the distribution of the fat, central or peripheral, through the analysis of the waist/hip ratio (WHR) [17]. Family history for early cardiovascular events were analyzed as previously reported [18].

We also recruited 67 normotensive controls aged 40 to 55 years. Written informed consent was obtained from all the subjects enrolled in this study and this study was approved by Ethic Committee of our Institution.

Exclusion criteria included secondary hypertension, endocrinal disease, cardiovascular diseases (defined as myocardial infarction and recent stroke within previous 6 months, heart failure) severe chronic renal failure, alcoholism and psychiatric problems. All patients were subsequently divided into two groups according to the presence of target organ damage such as left ventricular hypertrophy, carotid atherosclerosis, microalbuminuria, retinopathy and diabetes mellitus. At the time of enrolment, Blood Urea Nitrogen (BUN), creatinine and clearance, glycaemia, electrolytes (serum sodium, potassium, chloride), were measured for each patient by routine laboratory methods. Genotyping was performed by investigators blinded to clinical status.

### Genotype determination

Genomic DNA was prepared from blood leukocytes by established methods (kit Promega). The presence of the T-786C and G894T eNOS polymorphisms was examined by PCR-RFLP analysis. The introne 4 polymorphism was investigated by voltage gradient gel electrophoresis (VGGE). To ensure objectivity, DNA analysis was blinded to clinical status of the patients.

A set of primers was designed to amplify a 206 bp fragment including the missense Glu298Asp variant (G894T polymorphism) [5'-CAT GAG GCT CAG CCC CAG-3'(foward) and 5'-AGT CAA TCC CTT TGG TGC TCA C-3' (reverse)]. The PCR fragment digested overnight at 37°C with the *Mbo*I restriction enzyme was separated by electrophoresis on 2% agarosium gel and visualized by etidium bromide staining. In the presence of a T nucleotide at position 894 corresponding to Asp 298, the 206 bp PCR product was cleaved into two fragments of 119 and 87 bp; whereas the presence of a G removed the restriction site recovering a single fragment of 206 bp. The T-C transition at position 786 in the 5' flanking region of eNOS gene was investigated by PCR using 5'-ATG CTC CCA CCA GGG CAT CA-3' (foward) and 5'-GTC CTT GAG TCT GAC ATT AGG G-3' (reverse), as primers. The PCR fragment (236 bp long) was digested with the *NgOA*IV restriction enzyme overnight at 37°C. The wild type (786T) has no cleavage site, whereas in the presence of 786C the PCR product is cleaved into two fragments of 203 and 33 bp. The 27-bp repeat polymorphism in introne 4 was investigated by 5'-AGG CCC TAT GGT AGT GCC TTT-3'(forward) and 5'-TGC TCC TGC TAC TGA CAG CA-3'(reverse) as primers. Two fragments of different length were obtained: one of 220 bp corresponding to the allele b with five repetitions of 27 couples of bases and one of 193 bp (allele a) with four repetitions. Voltage gradient gel electrophoresis (VGGE) discriminate with a good resolution the two fragments on a 2% agarosium gel. In fact, 4bb-carriers produce a visible single band of 220 bp; 4ba- carriers two bands, one for the allele b (220 bp) and one for the allele a (193 bp); 4aa-carriers a single band of 193 bp.

### Albumin Excretion Rate (AER)

To eliminate the intra-individual day-to-day variability of AER, three consecutive 24 h urine collections were used. In addition, to assess the completeness of a 24 h urine collection, measurements of urinary rate of clearance of creatinine were evaluated. AER was measured by immunonephelometric assay (Bohering Institute; limit of detection, 0.1 mg/dl; Inter-assay coefficient 3.5%). Patients were defined as microalbuminuric if the level of AER was ≥ 20 and < 300 mg/24 h.

### Echocardiographic Measurements

All patients underwent an echocardiography examination M and B-mode, by a computerized echocardiography (ESAOTE, Italy) for the determination of the following parameters: 1) left ventricular telediastolic internal diameter (LVIDd), 2) interventricular septum (IVSTd), and 3) posterior wall thickness (PWTd). The Penn convention was used to calculate left ventricular mass (LVM). LVM was normalized for height to the 2.7 power [19]. Accordingly, all the hypertensives with LVM/h^2.7 ^≥ 50 g/m^2.7 ^for men and ≥ 47 g/m^2.7 ^for women were considered to have left ventricular hypertrophy (LVH). The relative wall thickness (RWT) [(PWTd/LVIDd)x2] was also calculated. Ejection fraction from left ventricular end-diastolic and end-systolic volumes was measured from the apical four chamber view, using the ellipsoidal single-plane algorithm. Mean ejection fraction was automatically calculated by the echocardiographic processing system. In our laboratory the ejection fraction calculated over five consecutive beats permitted optimal reproducibility and accuracy.

LV relaxation and filling were evaluated by pulsed-wave Doppler interrogation of the LV inflow tract from the apical four-chamber view, with the sample volume placed at the tips of the mitral valve. After a stable signal of the transmitral flow velocity was obtained, the Doppler cursor was moved toward the LV outflow tract in the apical five-chamber view for recording both mitral and aortic signals, including the closing click of the aortic valve and the opening click of the mitral valve. Doppler signals were recorded at high speed (80–120 mm/s) with the subjects in held expiration. An average of five beats was used for analysis.

Isovolumic relaxation time (IVRT) was calculated as the time from the closure click of the aortic valve to the opening click of the mitral valve. When either the closing or opening click was not identified, the time from the end of the aortic flow to the onset of mitral flow from the continuous wave interrogation of the LV inflow-outflow tract was used. Peak early transmitral flow velocity (E), peak late transmitral flow velocity (A), and the deceleration time of E velocity (DTE) were measured at the tips of mitral leaflets at the maximum amplitude of E velocity. DTE was measured as the time from peak E velocity to the time when E wave descent intercepts the zero line.

### Carotid evaluation

Arterial carotid wall was evaluated with high resolution B-mode ultrasonography with an ultrasound device (TOSHIBA SSA 270HG) equipped with a linear (7.5 MHz) transducer. Subjects were examined in the supine position with slight hyperextension of the neck on a longitudinal two-dimensional ultrasound image of carotid, the near and far arterial wall are displayed as two bright white lines separated by hypoechogenic space. The distance between the leading edge of the first bright line on the far wall (lume-intima interface) and the leading edge of the second bright line (media-adventitia interface) indicates the intima-media thickness of the far wall. A 1.5 cm segment of the common carotid artery (immediately caudal to the carotid bulb) and the proximal 1.5 cm segment of the internal carotid artery was considered. Within each segment, three measurements of IMT were taken ad wall thickness was not measured at the site of a discrete plaque.

### Statistical analysis

Data are reported as median (lower-upper quartiles) Comparisons of continuous variables were performed with the Mann-Whitney non parametric test. Frequencies were calculated using the comparison of two independent binomial proportions according to Armitage and Berry [20]. The Fisher test was used for the analysis of tables of contingency. Moreover, to study the possible associations among the examined variables and the cardiovascular damage, a model of logistic regression was built according to Hosmer and Lemeshow [21] for the calculation of adjusted odds ratios (OR) and intervals of confidence (IC). A p values <0.05 was considered statistically significant.

## Results

The clinical characteristics of the studied population was reported in table 1. Hypertensive and normotensive groups were homogeneous for sex, BMI, WHR. Age (p < 0.002), systolic (p<0.001); diastolic (p<0.001); mean blood pressure (p<0.001), left ventricular mass indexed for height^^2.7 ^(p < 0.004) and AER (p < 0.05) were significantly higher in hypertensives than normotensives. The prevalence of the clinical characteristics and the comorbidities in the hypertensives and normotensives was reported in table 2. In particular, a significant higher prevalence of family history for early cardiovascular events (p < 0.04), retinopathy (p < 0.001) left ventricular hypertrophy (p < 0.04) and carotid atherosclerosis (p < 0.001) was found in hypertensives than normotensives. The genotype frequency distribution of the three polymorphisms was reported in table 3. Out of 194 subjects, the G894T, T786C and introne 4 genotypes were determined for 194, 145 and 150 patients, respectively. Genotype frequency distribution is in accordance with the Hardy-Weinberg equilibrium and it is similar in the two groups. Only 4aa genotype distribution was significantly (p < 0.005) higher in hypertensives than normotensives.

**Table 1:** Clinical and biochemical characteristics of the studied population

	**Normotensives**	**Hypertensives**	***p *<**
Patients (n°)	67	127	
Women (%)	58	56	
Men (%)	42	44	
Age (years)	48.5 (40.0–55.0)	50.0 (44.0–57.0)	*0.002*
Weight (Kg)	77.4 (66.0–85.0)	80.0 (70.0–90.5)	*n.s*.
High(cm)	1.7 (1.6–1.7)	1.7 (1.6–1.7)	*n.s*.
BMI (Kg/m^2^)	27.1 (25.5–31.2)	29.3 (26.8–32.4)	*n.s*.
WHR	0.9 (0.9–1.0)	0.9 (0.9–1.0)	*n.s*.
SBP (mmHg)	127.5 (115.0–130.0)	140.0 (130.0–150.0)	*0.001*
DBP(mmHg)	80.0 (70.0–85.0)	90.0 (80.0–95.0)	*0.001*
MBP(mmHg)	94.2 (86.7–100.0)	106.7 (98.8–113.3)	*0.001*
BUN (mg/dl)	36.0 (31.0–39.0)	37.5 (33.3–44.0)	*n.s*.
Creatinine (mg/dl)	0.8 (0.7–0.9)	0.8 (0.7–0.9)	*n.s*.
Clearance (ml/min)	126.0 (100.3–153.0)	112.0 (88.1–140.0)	*n.s*.
AER(mg/24 h)	53.4 (21.3–150.5)	73.6 (44.4–122.0)	*0.05*
LVM (g)	145.8 (118.2–176.8)	161.1 (143.4–192.0)	*0.007*
LVM/h^2,7^	35.7(31.7–43.0)	41.4 (36.0–49.5)	*0.004*
Insulin (μUI/ml)	8.0 (4.5–12.4)	10.5 (8.0–15.0)	*n.s*.

BMI: body mass index; WHR: waist/hip ratio; PAS: systolic artery pressure; PAD; diastolic artery pressure; PAM: media artery pressure; BUN: blood urea nitrogen; AER: albumin excretion rate; LVM: left ventricular mass; LVM/h^2.7^: left ventricular mass/high^2.7^.

Data are presented as median (lower-upper quartiles)

**Table 2:** Prevalence of the clinical characteristics and the comorbidities in hypertensives and normotensives

	**Normotensives n.67**	**Hypertensives n.127**	***p *<**
Family history for early CV events (%)	0.0	8.6	*0.04*
Family history for not early CV events (%)	36.8	52.4	*n.s*.
Family history for diabetes mellitus (%)	13.2	15.2	*n.s*.
Diabetes mellitus (%)	3.0	5.5	*n.s*.
Retinopathy (%)	23.3	68.4	*0.001*
LVH (%)	13.9	28.7	*0.04*
Carotid Atherosclerosis (%)	20.6	51.3	*0.001*
Normal weight (%)	21.6	10.2	*n.s*.
Overweight (%)	46.0	43.9	*n.s*.
Obesity (%)	32.4	45.9	
Central obesity (%)	71.4	80.0	*n.s*.

CV = cardiovascular; LVH: left ventricular mass.

**Table 3:** Genotype frequency distribution of the three polymorphisms

		**Normotensives n. 67**	**Hypertensives n. 127**	***p *<**
G894T	GG	(19/67) 28%	(45/127) 35.4%	*n.s*.
	GT	(41/67) 61%	(70/127) 55.1%	*n.s*.
	TT	(7/67) 11%	(12/127) 9.5%	*n.s*.
	GT+TT	72%	61.6%	*n.s*.
Alleles		N°134	N°256	
	G	(79/134) 59%	(160/256) 63%	*n.s*.
	T	(55/134) 41%	(94/256) 37%	*n.s*.

				

		**Normotensives n. 38**	**Hypertensives n. 107**	***p *<**

T786C	TT	(8/38) 21%	(25/107) 23%	*n.s*.
	TC	(19/38) 50%	(51/107) 48%	*n.s*.
	CC	(11/38) 29%	(31/107) 29%	*n.s*.
	TC+CC	79%	78%	*n.s*.
Alleles		N°76	N°214	
	T	(35/76) 46%	(101/214) 47%	*n.s*.
	C	(41/76)54%	(113/214) 53%	*n.s*.

				

		**Normotensives n. 31**	**Hypertensives n. 119**	***p *<**

introne 4	bb	(24/31) 77%	(86/119) 72.5%	*n.s*.
	ba	(7/31) 23%	(30/119) 25%	*n.s*.
	aa	(0/31) 0%	(3/119) 2.5%	*0.005*
	ab+aa	23%	27.5%	*n.s*.
Alleles		N°62	N°238	
	b	(55/62) 89%	(202/238) 85%	*n.s*.
	a	(7/62) 11%	(36/238) 15%	*n.s*.

T894, C786 and 4a were considered rare alleles and their relationship with study variables was analysed by dominant or recessive models, presenting only the former (GG versus GT+TT; TT versus TC+CC; bb versus ab+aa) because the results did not differ. Accordingly, in table 4 the relationship of cardiovascular damage and genotypes in hypertensives and normotensives was reported. The subgroup with cardiovascular damage was characterized by a greater prevalence (p < 0.001) of GT+TT, TC+CC genotype and homozygosis for the allele b (table 4). Moreover, to appraise the role of hypertension in the association among eNOS variants and cardiovascular damage, we compared the prevalence of eNOS genotype in the hypertensives and normotensives both with cardiovascular damage (table 5). Hypertensives with cardiovascular damage were characterized by a greater prevalence of G894T polymorphism (p < 0.001) and introne 4 mutations (p < 0.05) than normotensives.

**Table 4:** Prevalence of cardiovascular damage according to genotype in all subjects (hypertensives and normotensives subjects)

**G894T**	**n. 104**	**%**	***p *<**
GG	38	36.5	0.001
GT+TT	66	63.5	
			
**T786C**	**n.90**		
TT	19	21.1	0.001
TC+CC	71	78.9	
			
**Introne 4**	**n.96**		
bb	72	75	0.001
ab+aa	24	25	

**Table 5:** Prevalence of eNOS genotype in hypertensives and normotensives with cardiovascular damage

**G894T**	**Normotensives n.67**	**%**	**Hypertensives n. 127**	**%**
GG	5	7.5	33	26.0*
GT+TT	14	20.9	52	40.9#

				

**T786C**	**Normotensives n.38**	**%**	**Hypertensives n.107**	**%**

TT	2	5.3	17	15.9
TC+CC	14	36.8	57	53.2

				

**Introne4**	**Normotensives n. 31**	**%**	**Hypertensives n. 119**	**%**

bb	10	32.3	62	52.1
ab+aa	3	9.7	21	17.6 ^§^

* p < 0,001 vs normotensives

*# *p < 0,007 vs normotensives

^§ ^p < 0,05 vs normotensives

### Logistic regression

We built a model of multiple logistic regression that included, among the independent variables, the presence or the absence of hypertension, BMI, age, sex and the heterozygous or homozygous genotypes The aim was to evaluate the possible association between the presence of cardiovascular damage and a determined eNOS genotype. From the analysis of the logistic regression, according to the model, only the age was associated to the presence of cardiovascular damage (OR 1.11; IC 95% 1.06–1.18).

## Discussion

Despite several clinical and experimental studies indicate a relevant role in the alteration of NO metabolism in the occurrence and severity of hypertension, no univocal date was reported on the influence of NO Synthase polymorphism and hypertension and its sequelae [5]. To our knowledge, this is the first study that has been designed to investigate the influence of G894T, T786C and intron4b/a eNOS polymorphisms on cardiovascular damage in hypertensive subjects. It seems to indicate the following data:

-a) the distribution of the genotypes G894T and T786C is similar in hypertensives and normotensives, whereas the homozygous condition for the allele a of the introne 4 (4aa) is present in the 2.5% of the hypertensives, and it is completely absent in the normotensives;

-b) the subjects with cardiovascular damage were characterized by an increased prevalence of 4bb, 894 (GT+TT) and 786 (TC+CC) eNOS genotypes;

-c) a significant higher prevalence of G894T and introne 4 variants was found in hypertensives than normotensives with cardiovascular damage. In our opinion, this association doesn't reflect unknown differences in population ancestry between the case-control groups. We consider the probability of false positive inference attributable to population stratification rather small because the hypertensive and control subjects were recruited from an ethnically homogeneous population with no indication of a significant amount of a recent genetic admixture.

Although these data might suggest an association between eNOS polymorphisms and cardiovascular damage, logistic regression analysis indicates that only the age may be significantly associated with the cardiovascular damage in our population. Although few studies have so far addressed a similar issue, and all in clinical setting different from ours, this finding is consistent with results of the meta-analysis of Zintzaras et al [5] who point out that, despite there is an elevated genetic contribution in the control of arterial hypertension, complex mechanisms and other factors (e.g. age and smoking) interact one with another, and, in the long period, are likely to hide the real role of eventual genetic variations. In particular, a low statistical power might explain the low-entity association because our sample was empowered by its size to discriminate only allelic odds ratio not lower than 2. Moreover even though genetic linkage studies take advantage by focusing on relatively well-defined intermediate phenotypes (i.e. left ventricular hypertrophy, retinopathy, intima-media wall thickness etc....), the influence of multiple susceptibility genes with powerful environmental or gene-gene interactions makes it difficult to exclude confounding by some unmeasured factors [22,23]. Finally, the discrepancy by us observed among the possible presence of a greater prevalence of some genotypes in the hypertensives with cardiovascular damage in comparison to the normotensives and the results of the logistic regression emphasize some methodological biases in defining the role of genetic associations with a clinical condition, like cardiovascular damage, notoriously in progress and modifiable over the time. In our study this might be due to age of subjects we have recruited. Accordingly, to evaluate if this is the case, perspective studies including young hypertensive subjects and focusing on gene environment interactions as well as haplotype analysis are necessary.
